# The emotional and motivational costs of poorly delivered academic feedback

**DOI:** 10.3389/fpsyg.2025.1585447

**Published:** 2025-06-30

**Authors:** David Reed Akolgo, Al Robiullah, Gerardo Ramirez

**Affiliations:** Department of Educational Psychology, Ball State University, Muncie, IN, United States

**Keywords:** feedback, reactance, respect, hassle, reactance theory, autonomy-threat, self-determination theory

## Abstract

**Introduction:**

This study uses psychological reactance theory and self-determination theory to explore whether disrespectful or disorganized feedback affects student motivation and classroom engagement.

**Methods:**

A sample of 148 undergraduates read one of four vignettes describing professor feedback that varied by tone (respectful or disrespectful) and clarity (low or high hassle). After reading the email, students completed measures assessing their emotional reactions, perceptions of the professor, and willingness to participate in class.

**Results:**

Students who received disrespectful messages reported stronger negative emotions, lower trust in the professor, and reduced willingness to participate. High-hassle feedback also lowered engagement, particularly when combined with a respectful tone. Interaction effects indicated that hassle weakened the positive impact of respectful communication.

**Discussion:**

Students interpret tone and structure in feedback as signals of respect and fairness. When communication feels disrespectful or unnecessarily complicated, students may disengage or comply for the sake of appearances while withdrawing emotionally. These findings suggest that instructors can protect student motivation by using clear and respectful language, especially in digital formats where intent can be harder to interpret.

## Introduction

How professors communicate with students can directly influence their academic performance, motivation, and sense of connection in the classroom. Dahmani et al. (2024) highlight how a professor’s communication style can directly influence students’ motivation, engagement, and academic performance. Their study shows that when communication is clear, supportive, and respectful, students are more likely to perform better and feel connected to their instructors. In contrast, Estrela et al. (2024) bring attention to a communication gap that often exists in higher education. While professors in their study believed they communicated well, students did not fully agree, particularly regarding how professors listened, and gave feedback.

Feedback facilitates the student-teacher relationship (Sethi and Scales, 2020; Katz et al., 2021; Henderson et al., 2019). Effective feedback helps students understand how they are performing and what they can do to improve (Williams, 2024; Hattie and Timperley, 2007; Wiggins, 2012; although see Meyer et al., 2025 for an interesting counter example that students rarely use feedback). Decades of research have shown that feedback can significantly influence student achievement, but its impact can help or hinder learning depending on how it is delivered (Goller and Späth, 2024; Ferris, 2018). One meta-analysis found that roughly one-third of feedback interventions led to decreased performance, raising concerns about the risks of poor feedback delivery (Goller and Späth, 2024; Kluger and DeNisi, 1998). For example, thoughtfully crafted feedback can motivate and clarify, while poorly delivered feedback may discourage students or provoke resistance.

This study investigates how certain qualities of feedback delivered influence student reactions, particularly through the lens of psychological reactance. Reactance is the resistant response people have when they feel their freedom is threatened (Heatherly et al., 2023; Brehm, 1966). In the context of education, a negative reaction to feedback can undermine its effectiveness. Hence, it is important to identify what aspects of online feedback communication might trigger such reactance.

### Digitally mediated feedback

The shift toward online and hybrid learning in recent years has made digitally mediated feedback an everyday experience for students (National Center for Education Statistics, 2023). Over half of college students now take at least one class online, meaning a large portion of feedback is delivered via digital platforms (e.g., learning management systems or email) rather than face-to-face (National Center for Education Statistics, 2024). This mode of delivery brings both opportunities and challenges.

Digital tools allow instructors to provide timely, detailed comments regardless of location. However, the absence of in-person cues (such as tone of voice or body language) can make it harder to convey nuance and empathy in feedback. Students may misinterpret the intent or respectful tone of written comments, potentially perceiving well-intentioned critiques as harsh or personal slights (Rabbani and Husain, 2024; Voelkel et al., 2020; Okonofua et al., 2016). The increasing prevalence of online instruction makes it particularly important to examine how students react to feedback in digital contexts. As digital formats continue to dominate education, understanding how students interpret the tone and structure of online feedback has become increasingly important for supporting motivation and engagement.

### Respect and “hassle” in feedback

Two qualities of online feedback that may affect student responses are the level of respect perceived in the message and the level of “hassle” it imposes on the recipient. In this paper, we define respect as language that upholds the inherent worth of others regardless of performance or status (Subramani and Biller-Andorno, 2022; Sennett, 2003). Respectful feedback uses a constructive, considerate tone: it avoids condescension, sarcasm, or any implication that the student is intellectually or morally inferior. For instance, “I see you put a lot of effort into this and there are areas we can refine” maintains respect, whereas a comment like “It seems you didn’t even try to understand the material” could be seen as demeaning. Students tend to respond better to feedback that is encouraging, respectful, clear and constructive, and they react poorly to feedback perceived as rude, sarcastic, vague, overly directive, or patronizing (Brooks et al., 2024; Wisniewski et al., 2020; Lee et al., 2015). Feedback perceived as harsh has been shown to disrupt students’ ability to process information and can leave a lasting negative impression (Hendriks et al., 2022; Taggart and Laughlin, 2017). Even when the content is intended to be helpful, the way it is phrased can affect whether students view it as fair or motivating (Güner Gültekin, 2024).

We use the term hassle to describe barriers that make feedback difficult for a student to interpret or act on (Park et al., 2022; Wiggins, 2012). High-hassle feedback might provide unclear direction or unnecessary complexity, leaving the student unsure how to proceed (Bertrand et al., 2004). Examples of hassle include vague instructions (e.g., “improve your analysis” without further explanation), poor organization (feedback points buried in an unstructured block of text), unclear expectations (not explaining the criteria or standard the student is being held to), or inefficient communication (such as overly long emails that obscure the main points). These kinds of hurdles can frustrate students (Ackerman and Gross, 2010; Brookhart, 2011; Carless, 2006; Higgins et al., 2002; Park et al., 2022; Roscoe et al., 2017). Even if the content of the feedback is valid, a confusing or roundabout delivery can lead to disengagement (Park and Ramirez, 2022). In sum, a respectful tone and low-hassle clarity are hypothesized to make students more receptive to feedback delivered online, whereas a disrespectful tone or high-hassle presentation may trigger negative reactions.

### Theoretical grounding in SDT and PRT

Our approach is informed by two complementary theoretical frameworks: self-determination theory and psychological reactance theory. Self-determination theory (SDT) posits that people have basic psychological needs for autonomy, competence, and relatedness (Olafsen et al., 2025; Deci and Ryan, 2013). Satisfying these needs leads to optimal motivation and engagement, but thwarting them can result in defensiveness or disengagement (Howard et al., 2024; Cheon et al., 2016; Bartholomew et al., 2018).

Feedback plays an important role in this dynamic. When feedback is delivered in an autonomy-supportive way, for example, with a respectful tone and clear suggestions for improvement, it can bolster a student’s sense of competence and autonomy. By contrast, using language that is controlling, implies they have no choice, and questioning students’ competence can thwart their desire for competence and autonomy (Buzzai et al., 2021). Recent meta-analytic work supports this claim, showing that need-thwarting behaviors can weaken motivation, while autonomy-supportive interventions enhance intrinsic engagement (Howard et al., 2024; Johansen et al., 2024). Research on teacher communication demonstrates that controlling language or a harsh tone of voice from instructors has been shown to provoke defiant, oppositional reactions from students (Paulmann and Weinstein, 2023; Weinstein et al., 2020). That is, when students feel their freedom or self-worth is under assault, they are less likely to embrace the critique and more likely to push back or withdraw.

Psychological reactance theory (PRT) provides a lens for understanding this pushback. Originally formulated by Brehm (1966), PRT explains that when individuals perceive their freedoms are being threatened or curtailed, they experience an aversive motivational state called reactance (Plohl and Musil, 2023). Reactance is a surge of resistance, a drive to reassert one’s autonomy.

As summarized in Table 1, both psychological reactance theory (PRT) and self-determination theory (SDT) explain how individuals respond when their autonomy is threatened. From the perspective of PRT, psychological reactance has both emotional and behavioral manifestations. Emotionally, a person in a state of reactance often feels irritation or anger at the source of the threat (Pop et al., 2025; Steindl et al., 2015a, 2015b). Behaviorally, they may resist by doing the opposite of what is urged, refusing to comply, or disengaging from the interaction (Clayton, 2022). In contrast, SDT emphasizes how autonomy frustration and unmet needs and relatedness for competence, needs for competence and relatedness, can also lead to negative outcomes such as disengagement, anxiety, and amotivation (Deci and Ryan, 2013; Howard et al., 2024). While PRT focuses on the restoration of threatened freedom, SDT highlights the importance of creating supportive environments that prevent such threats in the first place. This dual perspective is demonstrated in a recent study by Li and Liu (2025), which found that parental psychological control, an autonomy-threatening behavior, was associated with both increased psychological reactance and problematic smartphone use among adolescents. Their findings show how reactance can mediate the link between controlling environments and maladaptive behaviors (a PRT pathway), while also reinforcing SDT’s view that unmet psychological needs contribute to disengagement and poor self-regulation.

**Table 1:** Comparison of key aspects of psychological reactance theory (PRT) and self-determination theory (SDT).

Aspect	Psychological reactance theory (PRT)	Self-determination theory (SDT)
Definition	A motivational state triggered by perceived threats to one’s freedom or autonomy.	A macro-theory of motivation emphasizing the satisfaction of three basic psychological needs: autonomy, competence, and relatedness.
Theory’s focus	Restoration of threatened freedom.	Support and fulfillment of psychological needs to foster well-being and intrinsic motivation.
Common triggers	Controlling language, restrictions on choice, forced compliance.	Need-thwarting environments: controlling teaching, excessive evaluation, neglect of voice.
Emotional indicators	Anger, irritation, frustration.	Amotivation, anxiety, disengagement when needs are thwarted; vitality and interest when needs are supported.
Cognitive indicators	Counterarguing, negative evaluation of communicator or source of control.	Internalization or rejection of values depending on need support.
Behavioral responses when thwarted	Refusal to comply, defiance, doing the opposite of what is instructed.	disengagement, lack of persistence, or withdrawal
Strategies to reduce negative outcomes	Use of autonomy-supportive language, offering meaningful choice, validating perspectives.	Creating environments that support autonomy, competence, and relatedness.

In the context of student feedback, a student who perceives an instructor’s comment as unfairly controlling or disrespectful might react with resentment and choose to ignore the suggestions (Ryan and Henderson, 2018). This reaction is supported by findings from Yang et al. (2023), who observed that students often responded with anger, mistrust, or emotional disengagement when feedback was perceived as unreasonable or failed to meet their expectations, sometimes leading them to resist or dismiss the feedback altogether. This response is especially likely when students encounter controlling language or feel forced to take actions they do not fully endorse, especially among students with lower cognitive ability or low motivation, who are more likely to disengage from feedback altogether (Meyer et al., 2025; Steindl et al., 2015a, 2015b). Instructors who use coercive or disrespectful language may inadvertently trigger this kind of resistance, as such behaviors, like shouting, mocking, or public criticism, can be perceived as violations of respect and recognition, ultimately undermining students’ engagement (Mostafaei Alaei and Forough Ameri, 2021; Orejudo et al., 2020; Miller et al., 2007; Dillard and Shen, 2005).

Logistical barriers can also amplify reactance. Feedback that blocks students’ goals, is time-consuming to discern or act on may be perceived as an unjustified hassle (Park and Ramirez, 2022). Students already under stress may view these obstacles as a sign of faculty indifference or rigidity, further reducing their motivation to comply. These dynamics have been observed in feedback settings and also in broader educational systems, including registration processes, learning platforms, and financial aid interactions (Gellman and Meyer, 2023; Hove and Corcoran, 2008; Naveh et al., 2012; Reeves, 2015).

Although self-determination theory and psychological reactance theory have typically been applied in separate research traditions, several scholars have noted their conceptual overlap, particularly in how individuals respond to threats to autonomy. Reactance can be viewed as a motivational signal that emerges when autonomy needs are actively thwarted (Van Petegem et al., 2015, 2023; Soenens and Vansteenkiste, 2010). In this view, self-determination theory explains the broader framework of need satisfaction and frustration, while Reactance Theory offers a specific mechanism for how individuals respond when those needs, especially autonomy, are undermined. By combining these frameworks, we situate reactance not only as a defensive state but also as a consequence of disrupted motivational processes.

### Purpose of the present study

As more feedback is delivered online, understanding how its tone and clarity shape student reactions has become increasingly important for supporting learning. The present study aims to examine how online feedback dynamics, specifically the respectfulness of the tone and the presence of hassle factors, influence students’ emotional and behavioral responses.

We ask: Do students become less receptive (or even oppositional) when an instructor’s feedback comes off as disrespectful or needlessly difficult to interpret? And conversely, does a respectful, clear feedback style promote better uptake and attitudes? Our hypothesis is that disrespectful or high-hassle feedback threatens students’ sense of autonomy and worth, leading to reactance (e.g., feelings of anger, reduced willingness to follow the suggestions).

## Method

### Participants and data collection platform

We recruited participants using Prolific, an established online platform widely used in academic research for obtaining high-quality data from diverse populations (Peer et al., 2023). This platform has been successfully employed in psychological studies examining user behavior and emotional reactions to automated systems (Heatherly et al., 2023).

A study listing was created providing detailed information about the research project, its objectives, and the expected time commitment. The listing included specific eligibility criteria, such as being a college student aged 18 and above. N = 158 students signed up and completed the study. Ten participants were excluded due to missing data, failure to engage with the bot check, or not confirming college student status, resulting in a final sample of 148.

### Design and manipulation

Our study followed a 2(respect: high, low) x2(hassle: high, low) between-subjects design. Participants read a vignette portraying hypothetical scenarios wherein they imagined receiving email feedback from a professor after submitting an assignment. Importantly, participants were not required to read actual professor emails but were instead presented with imaginative vignettes. The vignettes described the professor’s feedback varying along two dimensions: Level of hassle (high, low) and Respect (high, low). See Appendix A.

The high hassle situation described a professor sending an email with a substantial block of unstructured and disorganized text, and unclear requests. Conversely, low hassle vignette scenarios exhibited the opposite attributes. The low respect situation described a professor sending an email that had harsh and critical language and used a negative and discouraging tone, explicitly pointing out mistakes without offering constructive feedback. The professor in this vignette was described as emphasizing weaknesses without acknowledging the student’s efforts or progress. The high respect vignette, in contrast, depicted an email characterized by positive and encouraging tones, constructive feedback, friendliness, and recognition of the student’s efforts and progress.

### Measures

Our assessment instruments aimed to gauge the student-teacher relationship, subsequent motivated behavior, and the formulation of realistic email responses to provided vignettes. Participants responded to a set of questions both before and after reading an email vignette depicting a professor’s feedback situation.

#### Pre-manipulation: general email behavior questionnaire

To ensure that any effects observed in the study were not due to pre-existing differences in how students typically interact with professors over email, we created a baseline measure of general email behavior. This questionnaire assessed participants’ typical responsiveness, attentiveness, and tone in email communication with professors. Sample items included: “I am responsive to emails from my professors,” “I am attentive to the content of emails I receive,” and “I am respectful in my tone and language when responding.” The specific questions were: “I am responsive to emails from my professors”; “I read email messages from your professors”; “I am attentive to the content of emails I receive from my professors”; “I am courteous in responding to emails from my professors”; “I promptly reply to emails from my professors”; “I read emails from my professors thoroughly before responding”; “I am respectful in my tone and language when responding to emails from my professors”; “I consider emails from my professors to be important.” Participants were asked to respond to these items on a scale from 1 (Strongly agree) to 4 (Strong disagree). The items were internally reliable (Cronbach’s alpha = 0.827).

#### *Post-manipulation measure*s

After participants were randomly assigned to one of four conditions and read the professor’s feedback vignette, we moved into the key phase of the study: measuring their perceptions of the professor. To guide analysis, we grouped the survey items into categories representing different aspects of the student-professor relationship. These included:

##### Perception of professor

To assess the student perception of their professor in class, we used a modified version of a scale originally developed by Okonofua et al. (2016). Our version of the scale used several items previously implemented and we included additional items that were conceptually designed to assess the extent to which they would respect their teacher. The scale measures seven items of students’ perception of their professor as; “This Professor deserves my respect”; “This Professor treats me fairly”; “I would address this professor in a respectful manner moving forward”; “Moving forward, I would have a positive relationship with the professor”; “This professor values my perspective and input”; “I would think this professor seems biased against me”; and “I would trust this professor’s expertise in the subject matter moving forward.” The items in this category were rated on a 4-point Likert scale, ranging from 1 (strongly disagree) to 4 (strongly agree). The items showed good reliability (Cronbach’s alpha = 0.94). Several related studies have used similar single-item measures (“Teachers and other adults at my school treat me with respect”) to evaluate students’ perceptions of respect following a teacher-focused intervention (Doyle et al., 2025; Gandhi et al., 2020).

##### Engagement and voluntary participation

Active class engagement plays a key role in deepening students’ understanding and strengthening the student-professor relationship. However, such engagement is often voluntary and tends to be higher when students feel respected by their instructors (Coristine et al., 2022). This section assessed participants’ willingness to engage in class following the email feedback vignette. We used a modified version of a scale (Okonofua and Ruiz, 2020; Okonofua et al., 2016) to measure students’ intentions to follow class rules, participate in discussions, stay motivated, and demonstrate appropriate classroom behavior. Sample items included: “I would be willing to follow rules in this class moving forward” and “I would be motivated to do well in class moving forward.” We also developed four additional items to assess voluntary academic effort not directly tied to grades, such as taking careful notes and contributing to class discussions. Participants rated each item on a 4-point Likert scale (1 = strongly disagree to 4 = strongly agree). The scale showed strong internal reliability (Cronbach’s alpha = 0.893).

##### Emotional responses

To assess participants’ emotional reactions to the professor’s email, we adapted items from the Positive and Negative Affect Schedule (PANAS), a well-established measure of affective states (Kumari et al., 2025; Watson et al., 1988). Participants were asked to indicate how likely they would be to experience each of the following emotions: annoyance, frustration, pleasure, disappointment, guilt, and confusion. Responses were rated on a 5-point scale from 1 (not at all) to 5 (extremely). The measure demonstrated strong internal reliability (Cronbach’s alpha = 0.90).

##### Resubmission behavior

Participants were asked about their expected response time to emails from professors. Response options ranged from within the hour to 36 h (approximately 1.5 days), with an additional option to select “I would not respond at all.” Furthermore, participants were asked about their likelihood of resubmitting assignments promptly, depending on the assignment’s point value (e.g., “if the assignment was worth very little points…” and “if the assignment was worth a lot of points….”). Responses were rated on a scale from 1 (extremely unlikely) to 5 (extremely likely). This measure was designed by the research team to examine how professor responses influence student behavior when the stakes are low versus high for resubmitting assignments.

##### Open ended response questions

Participants were asked to respond to two open-ended prompts. First, they were asked to describe in three sentences how they would behave differently in class after receiving the email. Second, they were instructed to write a realistic reply to the professor’s email, as they would in real life for that specific situation. These responses offered additional insight into how participants interpreted the tone and hassle level of the feedback. Open-ended responses are a valid method for obtaining nuanced information about participants’ thoughts and feelings, allowing for richer interpretations than closed-ended measures alone (Hansen and Świderska, 2024).

### Procedure

Participants began by completing the items measuring of general email behavior. All participants were then randomly assigned to one of four vignette conditions. Subsequently, participants responded to specific questions related to their perception of the professor, engagement and participation, academic performance, emotional response, willingness to respond to the email, and their approach in constructing a response after reading their assigned email vignette. The entire study took about 10 min to complete.

## Results

### Analytical framework

To improve interpretability, all items were reverse-coded where necessary so that higher scores consistently reflected stronger agreement. We then averaged items within each construct to create four composite variables representing key dimensions of the student-professor relationship. Results for these variables are summarized in Table 2 where we outline *F*-values. Corresponding means are presented in Table 3. We used factorial ANOVA to test the effects of the two manipulated factors, hassle and respect, on each outcome variable.

**Table 2:** Factorial ANOVA results for each individual items and their composite.

Post-assessment measures	Respect factor	Hassle factor	Interaction
Perception of professor	211.47***	6.01*	19.64***
Engagement and voluntary participation	54.78***	11.23***	4.43
Motivation	57.062***	8.37*	5.85*
Emotional	258.49***	29.31***	21.73***
Resubmission behavior	4.93	1.38	0.26

***p < 0.001; *p < 0.05.

Numbers represent F-values.

**Table 3:** Means and standard deviations of hassle factors and respect factors.

	Low hassle		High hassle	
Categories/dependent variables	Low respect	High respect	Low respect	High respect
	M (SD)	M (SD)	M (SD)	M (SD)
Perception of Professor (Overall):	2.09 (0.57)	3.65 (0.46)	2.25 (0.46)	3.08 (0.49)
This Professor deserves my respect	2.62 (0.82)	2.93 (0.83)	1.83 (0.61)	1.21 (0.47)
I would think this professor seems biased against me	2.44 (0.77)	2.06 (0.79)	3.19 (0.58)	3.50 (0.60)
This Professor treats me fairly	3.59 (0.69)	3.20 (0.76)	1.92 (0.65)	1.24 (0.49)
I would have a positive relationship with the professor	3.03 (0.63)	3.20 (0.85)	2.03 (0.65)	1.29 (0.52)
This professor values my perspective and input	3.12 (0.59)	3.30 (0.79)	1.97 (0.65)	1.41 (0.55)
I would address this professor in a respectful manner	2.12 (0.84)	2.20 (0.72)	1.72 (0.51)	1.32 (0.62)
I would trust this professor’s expertise	2.63 (0.77)	2.26 (0.62)	2.14 (0.64)	1.47 (0.60)
Engagement and Voluntary Participation:	2.73 (0.64)	3.59 (0.56)	2.61 (0.53)	3.09 (0.48)
I would be willing to follow rules in this class	2.26 (0.62)	2.15 (0.70)	1.89 (0.58)	1.39 (0.64)
I would exhibit appropriate behavior in this class	2.06 (0.69)	2.00 (0.68)	1.72 (0.51)	1.37 (0.68)
I would be willing to offer my perspectives	2.82 (0.80)	2.88 (0.91)	2.11 (0.58)	1.76 (0.79)
I would be motivated to do well in class	2.68 (0.73)	2.45 (1.00)	2.03 (0.61)	1.39 (0.60)
I would put in effort and participate in class	2.56 (0.71)	2.50 (0.88)	1.97 (0.61)	1.45 (0.69)
I would take careful notes in this professors class	2.03 (0.76)	2.20 (0.72)	1.89 (0.71)	1.53 (0.56)
Resubmission behavior				
I would re-submit the assignment relatively quickly	2.32 (0.84)	2.35 (0.86)	2.08 (0.69)	1.66 (0.71)
Resubmit the assignment (worth little points)	3.94 (1.48)	4.15 (1.73)	4.67 (1.12)	4.89 (1.1)
Resubmit the assignment (worth many points)	5.59 (0.61)	5.60 (0.71)	5.61 (0.80)	5.58 (0.72)
Emotional responses	3.47 (0.69)	1.36 (0.50)	3.54 (0.62)	2.38 (0.63)
Annoyed	3.82 (1.00)	3.75 (1.13)	2.44 (1.0)	1.29 (0.69)
Frustrated	4.24 (0.90)	4.24 (0.90)	2.58 (0.94)	1.45 (0.76)
Pleased	1.24 (0.65)	1.28 (0.82)	2.53 (1.08)	3.84 (0.95)
Disappointed	3.62 (0.92)	3.93 (1.19)	3.93 (1.19)	1.39 (0.72)
Guilty	2.03 (1.11)	2.10 (0.96)	1.28 (0.74)	1.32 (0.62)
Confused	2.80 (1.04)	2.23 (1.07)	3.28 (0.97)	3.76 (16)

### Pre-manipulation assessment: general email behavior

Our preliminary assessment examined students’ responses to general email behaviors. We anticipated uniform response patterns across conditions. There were no significant main effects or an interaction for either hassle or respect factor (*all ps > 0.05*), suggesting that students tend to uniformly comply with professor emails.

### Post-manipulation measures

As a reminder, after students received our fictitious email scenarios, they were asked to respond to a set of questions that probed their perception of the student-professor relationship, their willingness to engage in classroom instruction and behavior, their emotional reaction and subsequent communication with professors. We present data examining how our manipulation changed students’ perceptions here.

#### Perception of professor

**Figure 1:**
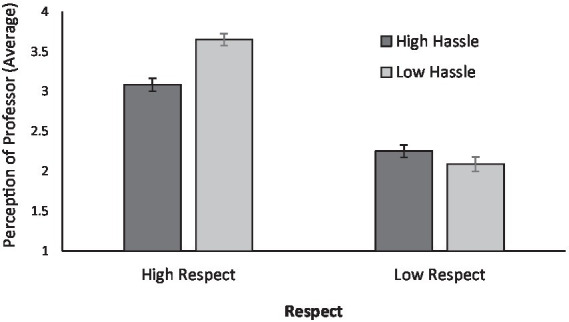
A bar graph depicting participants’ average perceptions of the professor by different levels of hassle and respect.

We first examined whether the level of respect and hassle influenced how students perceived their professor. This variable reflected the degree to which students held positive views of the professor, with higher scores indicating more favorable perceptions. A significant main effect of respect emerged, *F*(1, 144) = 211.47, *p* < 0.001, with participants in the high-respect condition (*M* = 3.37, *SD* = 0.55) rating their professor more positively than those in the low-respect condition (*M* = 2.16, *SD* = 0.53). There was also a smaller but significant main effect of hassle, *F*(1, 144) = 6.00, *p* = 0.015; participants in the low-hassle condition reported more favorable perceptions (*M* = 2.85, *SD* = 0.94) compared to those in the high-hassle condition (*M* = 2.68, *SD* = 0.63) (see Figure 1).

A significant interaction between respect and hassle was also found, *F*(1, 144) = 19.63, *p* < 0.001. In the high-respect condition, students exposed to low hassle (*M* = 3.65, *SD* = 0.46) perceived the professor more positively than those in the high-hassle condition (*M* = 3.08, *SD* = 0.49), *t* = −5.13, *p* < 0.01. However, no simple effects of hassle were observed within the low-respect condition (*p* > 0.05). These results suggest that hassle undermines perceptions of the professor when respect is high, but has little impact when respect is already low. See Tables 2, 3 for detailed results.

#### Engagement and voluntary participation

We next examined whether respect and hassle influenced students’ motivated behavior, specifically their willingness to engage and participate in class. A significant main effect of respect was found, *F*(1, 144) = 54.78, *p* < 0.001. Students in the high-respect condition (*M* = 3.35, *SD* = 0.58) reported greater engagement and willingness to participate than those in the low-respect condition (*M* = 2.67, *SD* = 0.59). A main effect of hassle also emerged, *F*(1, 144) = 11.23, *p* = 0.001, as well as a significant interaction between respect and hassle, *F*(1, 144) = 4.43, *p* = 0.037. In the high-respect condition, students who received low-hassle feedback reported higher engagement (*M* = 3.59, *SD* = 0.56) than those who received high-hassle feedback (*M* = 3.09, *SD* = 0.47), *t* (72) = 4.05, *p* < 0.01. In contrast, no simple effect of hassle was observed within the low-respect condition (*p* > 0.05). These findings suggest that even when feedback is respectful, poor organization or excessive demands can reduce students’ motivation to participate. See Tables 2, 3 for full results.

#### Resubmission behavior

Contrary to expectations, we did not find a significant effect of respect nor hassle, nor their interaction, in terms of how quickly participants said they would respond to professor emails (all *p*-values > 0.05). Similarly, there were no significant effects on students’ likelihood of resubmitting high-stakes assignments, suggesting that when grades are on the line, students tend to resubmit regardless of how they are treated.

However, a different pattern emerged for low-stakes assignments. A significant main effect of respect was found, *F*(1, 144) = 10.60, *p* < 0.001. Participants in the high-respect condition reported greater willingness to resubmit a low-point assignment (*M* = 4.78, *SD* = 1.06) than those in the low-respect condition (*M* = 4.05, *SD* = 1.60). This suggests that when the stakes are lower, respectful communication may play a more influential role in motivating student follow-through. In contrast, when the stakes are high, respectful communication played less of an influential role, perhaps because students feel they can’t enact their desire to engage in a reactant response. No other significant main effects or interactions were observed (all *p* > 0.05). See Tables 2, 3 for full results.

#### Emotional responses

We created a composite measure based on participants’ ratings of six emotional responses: annoyance, frustration, pleasure (reverse-coded), disappointment, guilt, and confusion. Higher scores indicated stronger negative emotional reactions to the professor’s email. A significant main effect of respect was observed, *F*(1, 144) = 258.50, *p* < 0.001, with participants in the low-respect condition reporting more intense negative emotions. A main effect of hassle also emerged, *F* (1, 144) = 29.31, *p* < 0.001, along with a significant interaction between respect and hassle, *F*(1, 144) = 21.73, *p* < 0.001. These patterns mirrored the results found for engagement and perceptions of the professor. Full results are presented in Tables 2, 3.

#### Students behavior change (open response)

As a reminder, we asked students to describe how they would behave in class after receiving the professor’s email. We coded these responses to identify the direction of behavioral change: approach orientation (increased effort or engagement), avoidance orientation (reduced effort or disengagement), or neutral/no change (Kappa = 0.97).

To examine how these behavioral intentions varied by the level of respect in the email, we conducted a chi-square test. The analysis showed no significant association between respect and behavior change (*χ^2^* = 1.49, *df* = 2, *p* > 0.05). Across both high and low respect conditions, the most common response was approach-oriented, with students reporting plans to increase their effort.

At first glance, this finding appears to contradict the survey data, which showed that students in the low-respect condition reported reduced engagement and willingness to participate in class. However, a closer look at the open-ended responses reveals a more complex pattern. Many students in the low-respect condition who expressed increased effort were not motivated by constructive engagement or a sense of collaboration with the professor. Instead, their responses reflected a defensive or performance-driven mindset. Students described behaviors such as taking more detailed notes, working harder on future assignments, or studying the professor’s expectations more closely, not out of respect, but as a strategy to avoid further criticism or to prove the professor wrong. For example, one student wrote:

“I would try to learn what they require of me, rather than what is necessarily right in terms of the content of the course. I would try to do more of my own research so that I feel well prepared with other assignments. I would probably be less willing to engage in conversation or discussion with the lecturer.”

This pattern suggests a form of strategic reactance: students may comply with academic demands to protect their grades or assert their competence, while emotionally distancing themselves and withdrawing from discretionary engagement like class discussion or informal interaction. Thus, although the behavior may look like increased engagement on the surface, it reflects a distinct psychological response from the authentic, reciprocal engagement observed in the high-respect condition.

The responses in the high respect condition reflected a more positive, collaborative dynamic. Students expressed openness to engaging with the professor, seeking clarification, and following instructions attentively. The predominant themes involved showing reciprocal respect, actively participating in class, and a willingness to implement the constructive feedback provided.

We then examined behavior change as a function of hassle level. A chi-square test revealed a marginally significant association between hassle and behavioral orientation (*χ^2^* = 5.63, *df* = 2, *p* = 0.06). In the low-hassle condition, students were more likely to report approach-oriented responses, such as planning to invest more effort or become more involved in class. For example, one student wrote, “I would pay more attention to my professor. I would attempt to make more of an effort to be involved. I would take more notes.”

In contrast, this pattern disappeared in the high-hassle condition, where approach, avoidance, and neutral responses were more evenly distributed. These results are consistent with the idea that excessive hassle can discourage proactive engagement, though the trend did not reach conventional levels of statistical significance.

#### Students email response

After reading the email vignette, participants were asked to compose a realistic reply to the professor, responding exactly as they would in real life for that situation. We coded these responses to assess how students navigate communication with authority figures under conditions of perceived disrespect or difficulty, providing insight into their emotion regulation and self-presentation strategies.

We developed a coding scheme based on thematic analysis to analyze the content of these email responses. Initial codes were generated by reviewing a subset of responses and identifying recurring patterns. Dependent variables included the presence or absence of boundary-setting language (e.g., “you cannot talk to me this way”), requests for clarification, use of honorific titles, and expressions of appreciation for feedback. These categories were selected based on preliminary coding and prior research on student-teacher communication, with a focus on how students assert themselves or preserve rapport.

Subsequent rounds of coding involved refining these categories and ensuring consistency across coders. Inter-rater reliability was high across all categories (Cohen’s kappa > 0.93), with discrepancies resolved through discussion until full consensus was reached.

We conducted a two-way ANOVA to examine the effects of respect and hassle on each coded response category. For all categories, we found no significant main effects or interactions were found for hassle (all *ps* > 0.05), nor were there significant interactions between respect and hassle (all *ps* > 0.05). Respect did not significantly affect the use of honorific titles or the frequency of clarification requests (both *ps* > 0.10).

However, there were marginal effects of respect on boundary-setting and appreciation. Participants in the low-respect condition were marginally more likely to include boundary-setting language (*M* = 0.20, *SD* = 0.40) than those in the high-respect condition (*M* = 0.098, *SD* = 0.30; *F*(1,144) = 3.20, P.076, partial η^2^ = 0.022), and marginally less likely to express appreciation (*M* = 0.66, *SD* = 0.48) compared to the high-respect group (*M* = 0.81, *SD* = 0.40; *F*(1,144) = 3.89, *p* = 0.051, partial η^2^ = 0.026).

Results suggest that, despite receiving disrespectful or disorganized feedback, most students maintained professional composure in their email responses. Only 16% of participants explicitly set boundaries, and even then, they did so with polite and measured language. For instance, one student in the high-hassle, low-respect condition wrote:

“Dear Professor Smith, Thank you for your feedback on my assignment. I appreciate your insights and suggestions for improvement. I would greatly benefit from clearer guidance on meeting the assignment requirements. Would it be possible to schedule a brief meeting to discuss this further? Regards”

In the rare instances where students did establish boundaries, they typically did so while maintaining politeness. As illustrated by this response from the low hassle, low respect condition:

"Dear Professor _____. Thank you for your email. Whilst I feel this email provided useful and clear guidance on the assignment, I am not happy with the feedback I have received. I feel it was harsh and did not offer anything constructive, and so I am very disappointed. Would it be possible to meet and discuss these concerns in person? Or is there someone else I can consult on the matter and gain a second opinion from? Many thanks."

These responses suggest that students may be strategically managing their communications to preserve their academic relationships, even when confronted with inappropriate professorial behavior. Discussion.

## Discussion

### Students’ responses to respectful vs. disrespectful feedback

The present findings highlight a divergence in student reactions depending on the tone of digitally delivered feedback (Zhang et al., 2025; Alsaiari et al., 2024; Ryan and Henderson, 2018). When students were given a vignette in which the professor feedback was communicated in a respectful manner, students reported more positive perceptions, milder emotional reactions, and a willingness to engage constructively with the comments. In contrast, disrespectful feedback triggered strong negative emotions and a drop in engagement (Hill et al., 2021). This finding is backed by work demonstrating that students commonly reject or disengage from feedback that is delivered with a negative tone or perceived lack of respect, even when the content is potentially useful (Lipnevich et al., 2025; Ryan and Henderson, 2018).

Data from the qualitative responses show a similar pattern. Students described feeling “demoralized,” “angry,” or “disheartened” when receiving feedback they perceived as rude or demeaning. Several students noted that respectful criticism, even when pointing out areas for improvement, felt “constructive” and “fair.” Disrespectful criticism, in contrast, felt like a personal attack. In low-respect scenarios, some students admitted they would shut down emotionally and stop caring about the assignment. Others, however, said they would have complied with the feedback only to avoid penalty or to get it done, not because they valued the input. These candid remarks were backed by the survey findings in which disrespect in feedback affected emotions and also alters how students choose to engage with their work (see Lang et al., 2022 for similar findings).

### Psychological reactance and student resistance

These outcomes, such as feeling demoralized, angry, or disheartened, withdrawing emotionally, or complying only to avoid penalties, can be understood through the lens of psychological reactance theory, which explains how people respond or push back when they feel their freedoms or autonomy are threatened (Frey et al., 2021; Yang and Kruschke, 2024; Brehm, 1966). Clayton et al. (2024) and Others have shown that threats to autonomy can elicit resistance through both direct and cognitive pathways (Clayton et al., 2024; Silvia, 2006). Others found that forceful, controlling language from instructors increased students’ perceived threat and reactance, often leading to disruptive or disengaged behaviors (found Robey, 2021; Ball and Goodboy, 2014). They also demonstrated that respectful, non-controlling language could reduce reactance, a finding consistent with our observation that high-respect feedback helped preserve student engagement.

The qualitative responses suggest that students may have expressed resistance in more than one way. Some described disengagement, such as ignoring the feedback or mentally checking out. Others described continuing to work hard but with a tone that implied frustration, defensiveness, or a desire to prove the professor wrong. This kind of strategic behavior resembles patterns observed by Ohtani and Yamamura (2024), who found that students sometimes comply on the surface while emotionally disengaging when exposed to controlling feedback. Similarly, Niemiec et al. (2022) describe strategic disengagement as a possible self-protective response in environments that frustrate autonomy. Our results cannot confirm such patterns directly, but the qualitative data are consistent with this interpretation. Even when students continue participating, disrespectful feedback may shift the nature of their motivation and reduce the depth of their engagement.

Within the qualitative responses, students described being more attentive or taking detailed notes not from intrinsic motivation but to perform better in future assessments or to demonstrate competence in spite of the professor’s tone (Bjørndal, 2020; Blunden and Brodsky, 2024). The fact that students continued resubmitting high-stakes assignments after receiving disrespectful or disorganized feedback demonstrates this pattern. Students appeared to differentiate between low-cost disengagement, such as avoiding voluntary participation, and high-cost compliance, such as submitting graded work (Rehfeldt et al., 2010; see also Curiel et al., 2023). These findings align with the principles of expectancy-value theory, which posits that motivation is influenced by the expected outcome and the value placed on that outcome (Feather, 2021).

Rather than representing a contradiction, this duality reflects a complementary pattern in how students restore autonomy. Students typically withdraw from optional activities like class discussions while simultaneously redirecting their efforts toward performance-based tasks directly affecting their grades. This strategic reallocation of engagement represents students’ practical response to academic environments, balancing personal autonomy needs against the realities of institutional evaluation structures and power dynamics. This selective engagement reflects a cost–benefit analysis rooted in the structure of the academic environment, where students must balance their need for autonomy with the reality of hierarchical power and high-stakes evaluation (Hill et al., 2021).

This strategic selectivity demonstrates how students navigate academic power dynamics. Many chose to compartmentalize their responses, avoiding unnecessary conflict while still meeting basic expectations. This was evident in their written replies to professors. Despite facing disrespect or excessive hassle, most students maintained a polite and professional tone in their email responses. Only 16 percent set explicit boundaries, and even those who did so framed their comments respectfully.

This restraint can be interpreted as a form of impression management or strategic self-protection within a hierarchical context (Blunden and Brodsky, 2024). The risks of confronting a professor, such as lowered grades or reputational harm, may outweigh the benefits. This helps explain why students often maintained a deferential tone, even when responding to inappropriate communication (Ohtani and Yamamura, 2024).

### Autonomy, motivation, and the need for respect

Another way to understand these findings is through self-determination theory (Wang et al., 2024; Deci and Ryan, 2013), which emphasizes the role of autonomy, competence, and relatedness in human motivation. Respectful feedback likely supports all three needs. It treats the student as a competent and autonomous partner in the learning process, and it maintains a sense of connection rather than alienation. Disrespectful feedback, by contrast, undermines autonomy by being controlling in tone, undermines competence by being disparaging, and undermines relatedness by signaling disconnection or condescension. According to Howard et al. (2024) and Deci et al. (2008), such frustration undermines motivation and well-being, often leading to disengagement and maladaptive behaviors. These outcomes are consistent with self-determination theory, which emphasizes that when students’ basic psychological needs, especially autonomy, are thwarted, they are more likely to experience emotional distress and reduced academic engagement. These patterns have been widely observed across educational settings, where controlling or need-thwarting teaching behaviors have been linked to declines in motivation, participation, and overall learning outcomes (Opdenakker, 2021; Koka, 2020; Cheon et al., 2016; Bartholomew et al., 2018). More recently, Zhang et al. (2024) demonstrated an association between teachers using autonomy-supportive language in their feedback and student need satisfaction.

In our study, disrespectful or controlling digital feedback may have triggered need frustration, thereby amplifying students’ psychological reactance and fueling both overt and covert resistance. Conversely, when feedback was respectful, students’ basic needs were more likely to be satisfied. They felt heard and trusted, which increased their willingness to act on feedback voluntarily.

This distinction is relevant because controlled and autonomous motivation are associated with different academic and emotional outcomes. Controlled motivation has been linked to lower persistence, negative emotions, and psychological strain (Yang et al., 2022), while autonomous motivation tends to support deeper engagement and better well-being (Santana-Monagas et al., 2025; Ryan and Deci, 2017). While we did not measure motivation types directly, the tone of feedback may plausibly shape students’ reasons for engaging. The qualitative responses point to this possibility, suggesting that respectful communication may foster more willing engagement, whereas disrespectful feedback may lead to surface compliance without internal commitment.

This distinction between willing participation and compliance under pressure has practical implications. Students may finish a task either because they feel motivated to grow or simply to meet expectations. That difference in mindset can shape both the quality of their learning and how they approach future feedback. A student who feels respected and self-motivated is likely to approach learning with openness and curiosity. A student who feels compelled by pressure or disrespect may do the work but miss the opportunity for growth.

This contrast was especially clear in how students described their reactions. In respectful conditions, students said they felt motivated to improve and appreciated the guidance. In disrespectful conditions, students described themselves as just trying to get it done or avoiding further criticism. The difference reflects a shift from internalized motivation to compliance under duress.

### The role of hassle

Although respect played a stronger overall role in shaping student responses, our findings suggest that hassle also matters, particularly when the tone of feedback is already respectful. In high-respect conditions, students responded more positively when the feedback was clearly organized and easy to follow, consistent with studies showing that students are more likely to engage with helpful resources when messaging is simplified and accessible (Bettinger and Long, 2017; Page et al., 2023). This suggests that clear and structured feedback can further enhance student motivation when delivered within a respectful relational context.

Conversely, when feedback lacked respect, even low-hassle conditions did little to improve students’ willingness to participate (Park and Ramirez, 2022). Our results align with research in public policy showing that individuals disengage from beneficial processes when they feel judged or mistreated, regardless of how user-friendly the system is (Pratt et al., 2023). Hassle-free feedback cannot compensate for a disrespectful tone; in such cases, students may already be emotionally withdrawn. These patterns suggest that respectful communication sets the foundation for how students interpret and respond to feedback, while structural clarity acts as a secondary enhancer when that foundation is in place. Without respect, clarity alone loses much of its motivational power.

Importantly, respect and hassle did not significantly affect students’ stated willingness to respond to email or resubmit high-stakes assignments. Regardless of the condition, most students maintained a professional tone in their responses. This restraint likely reflects a calculated decision to preserve academic relationships and avoid escalation, even when emotional frustration was present. While this restraint is understandable, it may also carry unintended consequences. By responding with extra effort or politeness rather than setting boundaries, students may unintentionally signal that disrespectful communication is acceptable. These dynamic risks reinforcing problematic behavior, making it more likely that professors will continue to use the same tone in future interactions.

These findings offer a clearer picture of how students interpret feedback in online academic environments. Respectful communication provides a foundation for engagement. Without it, even well-structured tasks lose their motivational value (Evans et al., 2024; Feldon et al., 2019). Conversely, when respect is present, reducing hassle can further support students’ willingness to participate and follow through on feedback. This interplay between tone and structure suggests that both elements influence student engagement, with respect potentially playing a foundational role.

### Implications

Receiving critical academic feedback can provoke strong emotional responses and threaten students’ sense of self-worth, particularly during their early years in college (Hill et al., 2021). Students may respond by disengaging from the learning process, minimizing the importance of the feedback, or justifying their performance in an effort to preserve self-image (Forsythe and Johnson, 2017; Ohtani and Yamamura, 2024). Our study adds to this literature by showing that feedback delivered in a disrespectful tone can intensify these reactions, reducing students’ motivation and willingness to engage.

Although our data centers on students’ responses, these responses are shaped by faculty behavior. Instructors, as the primary agents of feedback and evaluation, play a role in creating environments that either support or erode student motivation (Dahmani et al., 2024; Estrela et al., 2024). Our results suggest that communication strategies emphasizing respect, and clarity can foster better student engagement. When feedback is clouded by faculty frustration, tone can unintentionally become dismissive or harsh. Emerging technologies can aid faculty to regulate emotional expression in writing. For example, AI-driven platforms that assess tone and structure can help instructors revise their feedback to promote clarity and reduce unintended harshness (Florea and Croitoru, 2025; Lange and Parra-Moyano, 2025). Additionally, research on “wise feedback” shows that pairing critical comments with an affirmation of the student’s potential to meet high standards can significantly improve receptiveness, especially among marginalized groups (Yeager et al., 2014; Troy et al., 2024). This approach models how feedback can be both honest and respectful, reinforcing high expectations while preserving the student’s integrity.

At the same time, institutions must invest in helping students respond constructively to the emotional challenges of feedback. While some students eventually learn to depersonalize criticism and reframe it as constructive input (Hill et al., 2021), these skills often emerge only after repeated negative experiences. Rather than relying on trial and error, first-year programs can proactively foster feedback resilience through targeted support (Jackson et al., 2024). For example, Briscoe et al. (2023) found that students who participated in the “Grow Your Academic Resilience” workshop reported higher confidence and greater willingness to engage with instructor feedback. These brief sessions taught students how to normalize setbacks, regulate their emotions, and respond to criticism constructively.

Students can also benefit from reappraising that anger in professor emails as stemming from frustration at seeing potential squandered. For instance, Wang et al. (2023) found instructors often feel angry at students who are not meeting their potential. When instructors exert anger, it can be out of a genuine pedagogical concern which can actually enhance student engagement rather than diminish it. Another promising approach is self-affirmation (Easterbrook and Hadden, 2021; Cohen et al., 2009). Several studies demonstrated that brief reflective exercises focused on personal values can reduce defensiveness and buffer students from the psychological threat of negative feedback (Escobar-Soler et al., 2023). By helping students reinforce a sense of identity beyond academic performance, self-affirmation strategies make it easier to accept critique without internalizing it as a judgment of worth. Integrating these practices into orientation sessions or early coursework may help students approach feedback with more adaptive appraisals and resilience.

### Limitations and future directions

There are a number of limitations that need to be addressed. First, the use of hypothetical vignettes, while useful for experimental control, may not fully capture the complexity of real-life academic interactions. Future research could explore these dynamics in naturalistic settings, including real email exchanges and in-person feedback scenarios.

Additionally, future studies should go further into the duality of reactance responses observed in this study: the instinctual withdrawal to protect autonomy and the determination to engage more deeply. Exploring these contrasting reactions across varying academic stakes and contexts can offer valuable insights into the decision-making processes underlying reactance and the factors that determine which strategy students adopt.

Furthermore, future research should explore whether disrespect has become normalized in academic settings, as suggested by students’ tendency to maintain a respectful tone even after receiving disrespectful feedback. Qualitative studies or longitudinal research could help uncover the power dynamics involved and guide efforts to address these problems, creating environments that respect student autonomy and promote constructive engagement with feedback.

Future research should also investigate the professor’s perspective when delivering feedback. Disrespectful or disorganized emails may not always reflect a lack of communication skill, but rather, may stem from instructors’ emotional exhaustion and frustration at trying to reach students. Faculty often face repeated challenges such as missed deadlines, low-effort submissions, unresponsiveness, and general apathy from students (Farouk, 2010; Morris and King, 2018; Park and Ramirez, 2022). Over time, these patterns can contribute to burnout and increase the likelihood that feedback becomes terse, vague, or less constructive. Understanding these emotional pressures is critical to interpreting the tone of faculty feedback and identifying points of intervention.

Future research should therefore investigate the professor’s perspective more directly. Although many students in our study reacted negatively to perceived disrespect, instructors may be using these comments as a way to push students to recognize and address weaknesses (Rabani et al., 2024). This aligns with the concept of pedagogical anger, in which critical or stern communication is driven by concern for student learning (Wang et al., 2023). However, most research on pedagogical anger has focused on K–12 settings. College-level instruction introduces different norms around autonomy and professionalism, which may shape how faculty expressions of frustration are delivered and received. Investigating how faculty intent aligns or misaligns with student interpretation in higher education contexts remains an important direction for future work.

## Conclusion

This study provides new insight into how the tone and structure of online feedback shape students’ emotional responses, motivation, and engagement. Across both quantitative and qualitative measures, students were highly responsive to the respect conveyed in feedback, and to a lesser extent, the clarity and organization of the message. Disrespectful feedback eroded student-professor rapport and triggered psychological reactance, leading students either to disengage or to comply in ways that lacked authentic investment. At the same time, many students responded with professionalism and increased effort, even in the face of disrespect, an adaptive strategy that, while self-protective, may also reinforce unconstructive communication norms. These findings demonstrate the role of respectful interaction in higher education, especially in digital contexts where tone is more easily misinterpreted and opportunities for repair are limited. Future work should examine methods to support how faculty deliver feedback and the relational and structural qualities that shape how it is received and acted upon.

## Data Availability

The original contributions presented in the study are included in the article/supplementary material, further inquiries can be directed to the corresponding author.

## Appendix A. Vignette for the low respect-high hassle condition

It was a busy day for you, a dedicated student juggling your coursework. As you checked your email, you received a message from your Professor Smith, regarding an assignment that you submitted.

As you opened the email, you immediately noticed that it was one big giant block of text without any paragraphs or breaks, making it difficult to read and comprehend. The lack of formatting and organization made the email overwhelming and confusing to understand.

As you read through the email, you found that Professor Smith’s communication was disorganized. The email lacked clear headings or bullet points, and important information was buried within the lengthy text.

Professor Smith made multiple requests, but they were not clearly outlined, and you had to reread the email several times to grasp the main points being conveyed. The email also lacked specific guidance on how you could meet the requirements of the assignment, leaving you feeling uncertain and confused about what was expected of you.

Professor Smith used harsh and critical words, pointing out your mistakes and shortcomings without providing any constructive feedback or solutions for improvement on your assignment. He used a negative and discouraging tone, emphasizing your weaknesses, and offering no help. The email also had an unfriendly and unapproachable tone.
